# Molecular identification of badger-associated *Babesia* sp. DNA in dogs: updated phylogeny of piroplasms infecting Caniformia

**DOI:** 10.1186/s13071-018-2794-8

**Published:** 2018-04-11

**Authors:** Sándor Hornok, Gábor Horváth, Nóra Takács, Jenő Kontschán, Krisztina Szőke, Róbert Farkas

**Affiliations:** 1 Department of Parasitology and Zoology, University of Veterinary Medicine, Budapest, Hungary; 2 Veterinary Authority, Csurgó, Hungary; 3 Plant Protection Institute, Centre for Agricultural Research, Hungarian Academy of Sciences, Budapest, Hungary

## Abstract

**Background:**

Piroplasms are unicellular, tick-borne parasites. Among them, during the past decade, an increasing diversity of *Babesia* spp. has been reported from wild carnivores. On the other hand, despite the known contact of domestic and wild carnivores (e.g. during hunting), and a number of ixodid tick species they share, data on the infection of dogs with babesiae from other families of carnivores are rare.

**Methods:**

In this study blood samples were collected from 90 dogs and five road-killed badgers. Ticks were also removed from these animals. The DNA was extracted from all blood samples, and from 33 ticks of badgers, followed by molecular analysis for piroplasms with PCR and sequencing, as well as by phylogenetic comparison of detected genotypes with piroplasms infecting carnivores.

**Results:**

Eleven of 90 blood DNA extracts from dogs, and all five samples from badgers were PCR-positive for piroplasms. In addition to the presence of *B. canis* DNA in five dogs, sequencing identified the DNA of badger-associated “*Babesia* sp. Meles-Hu1” in six dogs and in all five badgers. The DNA of “*Babesia* sp. Meles-Hu1” occurred significantly more frequently in dogs often taken to forests (i.e. the preferred habitat of badgers in Hungary), than in dogs without this characteristic. Moreover, detection of DNA from this *Babesia* sp. was significantly associated with hunting dogs in comparison with dogs not used for hunting. Two PCR-positive dogs (in one of which the DNA of the badger-associated *Babesia* sp. was identified, whereas in the other the DNA of *B. canis* was present) showed clinical signs of babesiosis. Engorged specimens of both *I. canisuga* and *I. hexagonus* were collected from badgers with parasitaemia, but only *I. canisuga* contained the DNA of “*Babesia* sp. Meles-Hu1”. This means a significant association of the DNA from “*Babesia* sp. Meles-Hu1” with *I. canisuga*. Phylogenetically, “*Babesia* sp. Meles-Hu1” belonged to the “*B. microti*” group.

**Conclusions:**

This is the first detection of the DNA from a badger-associated *Babesia* sp. in dogs, one of which also showed relevant clinical signs. Based on the number of dogs with blood samples containing the DNA of “*Babesia* sp. Meles-Hu1” in this study (i.e. exceeding the number of *B. canis*-positives), these findings should not be regarded as isolated cases. It is assumed that dogs, which are used for hunting or frequently visit forests, are more likely to be exposed to this piroplasm, probably as a consequence of infestation with *I. canisuga* from badgers or from the burrows of badgers. The above results suggest that “*Babesia* sp. Meles-Hu1” should be added to the range of piroplasms, which are naturally capable of infecting hosts from different families of Caniformia.

## Background

Piroplasms (Apicomplexa: Piroplasmida) are unicellular, tick-borne parasites, which infect red and white blood cells of their vertebrate hosts [1]. Among them, *Babesia* spp. and *Theileria* spp. are the most significant from the veterinary point of view, in terms of both their geographical distribution and the range of affected host species.

In general, babesiae have long been regarded as host species-specific [2], but in the era of molecular biological methods the reported ranges of hosts susceptible to infection with a particular *Babesia* species have started to expand. When the currently known diversity of *Babesia* spp., infecting domestic carnivores as their typical hosts, is concerned, the number of relevant disease agents appears to be stable, despite the fact that during the past years the taxonomic rank and/or the naming of certain variants has changed or is still unresolved. Thus, in a worldwide context, dogs are susceptible to three species of the so-called large babesiae and three species of small babesiae [3].

However, an increasing diversity of *Babesia* spp. are reported from wild carnivores (e.g. in Japan: [4]), and the number of families within order Carnivora, in which piroplasms are molecularly detected, also increased (e.g. Ursidae [5, 6]; Herpestidae [7]; Hyaenidae [8]). Furthermore, mostly in individual cases, piroplasms from more distant host taxa might also occur in carnivores, as exemplified by *T. equi* or *B. caballi* in dogs (typical hosts being Equidae) [9, 10], or *T. capreoli* in grey wolves (typical hosts are Cervidae) [11]. Such occasional infections may even be associated with clinical signs in carnivores as “atypical hosts” [10].

These data highlight carnivores as a group important from the point of studying piroplasms, especially at the interface of domestic and wild carnivores, for which mutually infective *Babesia* spp. have been reported [12]. In the above context, the present study aimed to molecularly investigate piroplasms in a region of Hungary, where emerging tick species and tick-borne pathogens have been reported [13, 14]. To achieve this, blood samples and ticks were collected from dogs and badgers, consequently analyzed with PCR and sequencing.

## Methods

Blood and tick samples were collected from dogs and European badgers (*Meles meles*) originating from 20 locations (17 and 3 for dogs and badgers, respectively) in southwestern Hungary (Somogy county) between March and October (dogs) and January to April (badgers) of 2017. From 90 dogs, EDTA-anticoagulated blood samples were drawn under clinical conditions (after skin surface sterilization, with sterile instruments) by cephalic venipuncture. The majority of sampled dogs (*n* = 80) were selected randomly (from regular patients of the Veterinary Clinic in Csurgó) and appeared to be healthy, but ten showed at least one clinical sign relevant to babesiosis. Animal data (owner, sex, age, mode of keeping) were recorded. From five freshly road-killed badgers EDTA-anticoagulated blood samples were collected (with sterile needle and syringe) from the heart, shortly after being reported to the Veterinary Authority. EDTA blood samples were frozen at -20 °C until further processing. In addition, ixodid ticks were removed from all animals with pointed tweezers then transferred into 96% ethanol for storage in separate vials according to host individuals. Tick species were identified according to standard keys [15], and ticks of the subgenus *Pholeoixodes* according to [16].

DNA was obtained using the QIAamp DNA Mini Kit (Qiagen, Hilden, Germany) following the manufacturer’s instruction and using extraction controls to monitor cross contamination of samples in each run. DNA was extracted from 200 μl blood of 90 dogs and 5 badgers, as well as from 33 ticks collected from badgers. These ticks belonged to two species, i.e. *Ixodes canisuga* and *I. hexagonus* (subgenus *Pholeoixodes*), selected on the basis of literature data supporting their role in the transmission of *Babesia* spp. of the *B. microti* group [17, 18]. DNA was extracted from the ticks individually, with the same method as from the blood samples, but including an overnight digestion in tissue lysis buffer and proteinase-K at 56 °C, and incubation in lysis buffer for 10 min at 70 °C. DNA samples were finally taken up in 130 μl elution buffer.

DNA extracts from all 95 blood samples and 33 ticks were screened for the presence of piroplasms by a conventional PCR [19]. This PCR amplifies an approximately 500 bp fragment of the *18S* rRNA gene of *Babesia*/*Theileria* spp. with the primers BJ1 (forward: 5′-GTC TTG TAA TTG GAA TGA TGG-3′) and BN2 (reverse: 5′-TAG TTT ATG GTT AGG ACT ACG-3′) [20]. The 25.0 μl final volume of reaction mixture contained 5.0 μl template DNA, 1.0 U HotStar Taq Plus DNA Polymerase (5U/μl) (Qiagen, Hilden, Germany), 2.5 μl of 10× Coral Load PCR Buffer (15 mM MgCl_2_ included), 0.5 μl dNTP mix (10 mM), 0.5 μl of each primer (50 μM) and 15.8 μl distilled water. Cycling conditions consisted of an initial denaturation step at 95 °C for 10 min, followed by 40 cycles of denaturation at 95 °C for 30 s, annealing at 54 °C for 30 s and extension at 72 °C for 40 s. The final extension was performed at 72 °C for 5 min.

Each PCR was run with positive and negative controls (i.e. sequence-verified DNA of *Babesia canis*, and non-template reaction mixture, respectively). PCR products were visualized in 1.5% agarose gel. Negative controls and extraction controls remained PCR negative in all tests. Purification and sequencing (directly from the PCR product, using the forward primer and generating the sequence twice per sample) were performed from all piroplasm PCR-positive samples at Biomi Inc. (Gödöllő, Hungary). Sequences were aligned and compared to reference GenBank sequences by nucleotide BLASTn program (https://blast.ncbi.nlm.nih.gov). All blood samples, which yielded unusual results (i.e. the presence of DNA from a *Babesia* sp. not yet reported in the relevant host) were re-tested in duplicates, by repeating the whole procedure from the beginning (DNA extraction, PCR and sequencing). Representative sequences were submitted to the GenBank database under the accession numbers MG778912-MG778915. Phylogenetic analyses were performed with the Maximum Likelihood method and Tamura-Nei model, using MEGA 6.0.

Rates of PCR-positivity were compared with the Fisher’s exact test and differences were considered significant when *P* < 0.05.

## Results

Among blood DNA extracts, 11 of 90 samples from dogs, and all five samples from badgers were PCR-positive for piroplasms. Sequencing identified two *Babesia* spp. in dogs. In six dog blood samples the DNA of a *Babesia* sp. was present, which was formerly reported from European badgers (*Meles meles*) in Hungary and designated as “*Babesia* sp. Meles-Hu1” (GenBank: KX218234). This genotype also corresponded to “*Babesia* sp. badger type-A” reported from Spain (GenBank: KT223484) and the UK (GenBank: KX528553), with 100% (472/472 bp) and 99.8% (471/472 bp) identity, respectively. In addition, the DNA of *B. canis* was detected in five blood samples from dogs: in one case corresponding to “genotype A” (430/430 bp; 100% identity with KP835549), and in four samples to “genotype B” (430/430 bp; 100% identity with KP835550) formerly reported in Hungary. In the blood of all badgers the DNA of “*Babesia* sp. Meles-Hu1” was present, which had 100% (472/472 bp) identity with the above dog isolate.

Concerning the distribution of *Babesia* species in dogs according to their mode of keeping, the DNA of “*Babesia* sp. Meles-Hu1” occurred significantly (*P* = 0.0001) more frequently in dogs often taken to forests (5 of 12) than in dogs without this characteristic (1 of 78) (Table 1). Moreover, the presence of DNA from this *Babesia* sp. was significantly (*P* = 0.00008) associated with hunting dogs (4 of 6 were infected) in comparison with dogs not used for hunting (2 of 84) (Table 1). Taking into account the sampling time, the dog samples containing the DNA of different *Babesia* spp. also showed an uneven seasonal distribution: while the DNA of “*Babesia* sp. Meles-Hu1” was detected in samples collected in March (*n* = 3) and August (*n* = 3), the DNA of *B. canis* was present in samples obtained in May (*n* = 2) or September (*n* = 3). Importantly, two PCR-positive dogs (one yielding the sequence of the badger-associated *Babesia* sp., the other that of *B. canis*) showed clinical signs of babesiosis (fatigue, renal failure and/or anaemia, icterus).

**Table 1:** Results of molecular analyses

	No. of dogs according to mode of keeping			No. of badgers (*n* = 5)	No. of ixodid ticks	
	Hunting in forest (*n *= 6)	Frequently in forest (*n* = 6)	Other (*n* = 78)		*Ixodes canisuga* (*n* = 27)	*Ixodes hexagonus* (*n* = 6)
*“Babesia* sp. Meles-Hu1”	4 (66.7%)	1 (16.7%)	1 (1.3%)	5 (100%)	18 (66.7%)	0
*Babesia canis*	0	0	5 (6.4%)	0	0	0
*Babesia*-negative	2 (33.3%)	5 (83.3%)	72 (92.3%)	0	9 (33.3%)	6 (100%)

Altogether 19 ticks were collected from dogs, i.e. *Dermacentor reticulatus* (*n* = 10), *Ixodes ricinus* (*n* = 8) and *I. hexagonus* (*n* = 1); and 53 ticks from badgers, i.e. *I. canisuga* (*n* = 34), *D. reticulatus* (*n* = 7), *I. hexagonus* (*n* = 6), *I. ricinus* (*n* = 3), *I. kaiseri* (*n* = 2) and *Haemaphysalis concinna* (*n* = 1).

In the molecularly analyzed 33 *Pholeoixodes* tick samples, only the DNA of “*Babesia* sp. Meles-Hu1” was found. Interestingly, while engorged specimens of both *I. canisuga* (*n* = 27) and *I. hexagonus* (*n* = 6) were collected from badgers, which had this *Babesia* sp. in their blood (as shown above), none of the *I. hexagonus* specimens were PCR-positive, but 66.7% (18 of 27) of *I. canisuga* DNA extracts contained “*Babesia* sp. Meles-Hu1” (Table 1). This was a significant association of this piroplasm with *I. canisuga* (*P* = 0.0045).

**Fig. 1:**
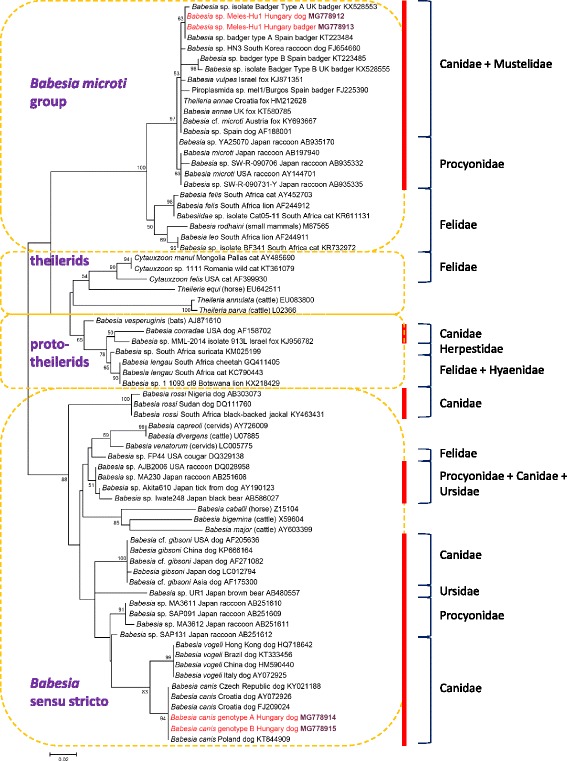
Maximum Likelihood tree of piroplasm DNA sequences reported from carnivores, shown according to their families. Sequences from this study are highlighted with red color. Piroplasm categories are named after [1] (except for *Theileria equi*, *Cytauxzoon* spp. and *Theileria* spp. united in one group). For Hyaenidae, the sequence KF270649 had a short coverage and therefore is not included (but in a sequence comparison it aligned with *B. lengau*). Vertical red columns mark adjacent families of Caniformia. The scale-bar indicates the number of substitutions per site

Phylogenetically, sequences of *B. canis* genotypes amplified from dogs in the present study clustered with other conspecific isolates of the clade “*Babesia* (*sensu stricto*)” (Fig. 1). On the other hand, sequences of “*Babesia* sp. Meles-Hu1” obtained from both dogs and badgers here belonged to the “*B. microti* phylogenetic group” (Fig. 1).

## Discussion

In this study, blood and tick samples collected from dogs and European badgers were molecularly analyzed for the presence of piroplasm DNA. The DNA from both *B. canis* and “*Babesia* sp. Meles-Hu1” were found. Based on literature reviews, in European badgers “*Babesia* sp. badger type-A” and “ *Babesia* sp. badger type-B”, as well as “B. annae” were reported [18]. In dogs as typical hosts, in a worldwide context, three small *Babesia* spp., i.e. *B. gibsoni*, “B. annae” (syn. “T. annae”, *B.* cf. *microti*) and *B. conradae*, as well as three large *Babesia* spp. (*B. canis*, *B. vogeli* and *B. rossi*) were hitherto known to occur [3] (Fig. 1). Rare accounts also attest the occasional finding of the DNA of piroplasms in dogs, for which the typical hosts are outside the order Carnivora (e.g. *B. caballi* [10]). However, this is the first molecular identification of the DNA from a badger-associated *Babesia* sp. in canine hosts. Taking into account the simultaneous occurrence of DNA from “*Babesia* sp. Meles-Hu1” in six dogs, one of which showed typical clinical signs of babesiosis, results of the present study suggest that the occurrence of “*Babesia* sp. Meles-Hu1” is not exceptional in dogs, and it might even have a pathogenic role in this host species.

The badger-associated *Babesia* genotype identified here corresponded to “*Babesia* sp. Meles-Hu1”, which was formerly identified in a badger in eastern Hungary (GenBank: KX218234), suggesting a widespread occurrence of this species in the country. *Babesia canis* genotypes “A” and “B” (GenBank: KP835549 and KP835550, respectively) were also reported formerly in Hungary from bats [21] and from questing *D. reticulatus* ticks [22].

The significantly higher number of “*Babesia* sp. Meles-Hu1”-positive samples among dogs frequently taken to forests (including hunting dogs) highlights the importance of the preferred habitat of badgers (i.e. forest in Hungary [23]) in the epidemiology of this infection. In particular, dogs in such environments, especially when getting into contact with badgers during hunting, may acquire infestation with *I. canisuga* from badgers or from the burrows of badgers.

On the other hand, all *B. canis*-positive dogs were identified in the sample group not associated with forests, most likely reflecting that the vector of this piroplasm (*D. reticulatus*) is an “open country tick species” [24] (in Hungary [25]). In addition, the time shift between PCR-positive samples containing either *B. canis* or “*Babesia* sp. Meles-Hu1” is also most likely a consequence of differences in the activity periods of their vectors.

*Ixodes canisuga* was proposed to be the vector of the badger-associated piroplasm, *B. missirolli* [17], and this vector potential was confirmed by the present results from the molecular analysis of badger ticks (*I. canisuga vs I. hexagonus*). Dogs were formerly reported to harbor *I. canisuga* in Hungary [26]. Such occasions would allow tick-borne badger-to-dog transmission. On the other hand, taking into account the usage of dogs during badger hunting in the evaluated region of Hungary, oral infection of relevant dogs with “*Babesia* sp. Meles-Hu1” cannot be completely ruled out. In support of this, (i) blood-borne transmission of babesiae was reported in fighting dogs (*B. gibsoni* [27]), and (ii) oral infection is possible in the case of the type-species of the phylogenetic group where “*Babesia* sp. Meles-Hu1” belongs (*B. microti* [28]).

Phylogenetic analysis showed that “*Babesia* sp. Meles-Hu1” identified in dogs belongs to the *B. microti* group, where piroplasms of the Mustelidae, Canidae and Procyonidae (suborder Caniformia) cluster together and are separated (with 100% support) from a phylogenetic group containing piroplasms of Feliformia (Fig. 1). Several examples attest that different species of carnivores within a family (e.g. Canidae) are susceptible to the same piroplasm (e.g. *B. canis*), while *Babesia* spp. are also known to infect carnivorous hosts from different families of Caniformia, as exemplified by high prevalences of *B. microti*-like infections in both Canidae and Procyonidae [12].

## Conclusions

This is the first detection of the DNA from a badger-associated *Babesia* sp. in dogs, one of which also showed relevant clinical signs. In this study, the number of dogs with blood samples containing the DNA of “*Babesia* sp. Meles-Hu1” exceeded the number of *B. canis*-positive samples, therefore these findings should not be regarded as isolated cases. It is assumed that dogs, which are used for hunting or frequently visit forests, are more likely to be exposed to this piroplasm, probably as a consequence of infestation with *I. canisuga* from badgers or from the burrows of badgers. These results suggest that “*Babesia* sp. Meles-Hu1” should be added to the range of piroplasms, which are naturally capable of infecting hosts from different families of Caniformia.

## References

[1] Schreeg ME, Marr HS, Tarigo JL, Cohn LA, Bird DM, Scholl EH (2016). Mitochondrial genome sequences and structures aid in the resolution of Piroplasmida phylogeny. PLoS One.

[2] Kuttler KL, Ristic M (1988). World-wide impact of babesiosis. Babesiosis of domestic animals and man.

[3] Kjemtrup AM, Kocan AA, Whitworth L, Meinkoth J, Birkenheuer AJ, Cummings J (2000). There are at least three genetically distinct small piroplasms from dogs. Int J Parasitol.

[4] Jinnai M, Kawabuchi-Kurata T, Tsuji M, Nakajima R, Fujisawa K, Nagata S (2009). Molecular evidence for the presence of new *Babesia* species in feral raccoons (*Procyon lotor*) in Hokkaido, Japan. Vet Parasitol.

[5] Jinnai M, Kawabuchi-Kurata T, Tsuji M, Nakajima R, Hirata H, Fujisawa K (2010). Molecular evidence of the multiple genotype infection of a wild Hokkaido brown bear (*Ursus arctos yesoensis*) by *Babesia* sp. UR1. Vet Parasitol.

[6] Ikawa K, Aoki M, Ichikawa M, Itagaki T (2011). The first detection of *Babesia* species DNA from Japanese black bears (*Ursus thibetanus japonicus*) in Japan. Parasitol Int.

[7] Leclaire S, Menard S, Berry A (2015). Molecular characterization of *Babesia* and *Cytauxzoon* species in wild South African meerkats. Parasitology.

[8] Williams B, Berentsen A, Shock B, Teixiera M, Dunbar M, Becker M (2014). Prevalence and diversity of *Babesia*, *Hepatozoon*, *Ehrlichiha* and *Bartonella* in wild and domestic carnivores from Zambia, Africa. Parasitol Res.

[9] Criado-Fornelio A, Martinez-Marcos A, Buling-Sarana A, Barba-Carretero JC (2003). Molecular studies on *Babesia*, *Theileria* and *Hepatozoon* in southern Europe - Part I. Epizootiological aspects. Vet Parasitol.

[10] Beck R, Vojta L, Mrljak V, Marinculić A, Beck A, Zivicnjak T (2009). Diversity of *Babesia* and *Theileria* species in symptomatic and asymptomatic dogs in Croatia. Int J Parasitol.

[11] Beck A, Huber D, Polkinghorne A, Kurilj AG, Benko V, Mrljak V (2017). The prevalence and impact of *Babesia canis* and *Theileria* sp. in free-ranging grey wolf (*Canis lupus*) populations in Croatia. Parasit Vectors.

[12] Alvarado-Rybak M, Solano-Gallego L, Millán J (2016). A review of piroplasmid infections in wild carnivores worldwide: importance for domestic animal health and wildlife conservation. Parasit Vectors.

[13] Hornok S, Horváth G (2012). First report of adult *Hyalomma marginatum rufipes* (vector of Crimean-Congo haemorrhagic fever virus) on cattle under a continental climate in Hungary. Parasit Vectors.

[14] Hornok S, de la Fuente J, Horváth G, Fernández de Mera IG, Wijnveld M, Tánczos B (2013). Molecular evidence of *Ehrlichia canis* and *Rickettsia massiliae* in ixodid ticks of carnivores from south Hungary. Acta Vet Hung.

[15] Babos S. Kullancsok - Ixodidea. Fauna Hungariae. 1965;18:1–38. (In Hungarian).

[16] Hornok S, Sándor AD, Beck R, Farkas R, Beati L, Kontschán J (2017). Contributions to the phylogeny of *Ixodes* (*Pholeoixodes*) *canisuga*, *I.* (*Ph.*) *kaiseri*, *I.* (*Ph.*) *hexagonus* and a simple pictorial key for the identification of their females. Parasit Vectors.

[17] Biocca E, Corradetti A (1952). *Babesia missirolli*, n. sp., parassita del tasso (*Meles meles*). Riv Parassitol.

[18] Barandika JF, Espí A, Oporto B, del Cerro A, Barral M, Povedano I (2016). Occurrence and genetic diversity of piroplasms and other Apicomplexa in wild carnivores. Parasitology Open.

[19] Casati S, Sager H, Gern L, Piffaretti JC (2006). Presence of potentially pathogenic *Babesia* sp. for human in *Ixodes ricinus* in Switzerland. Ann Agric Environ Med.

[20] Hornok S, Mester A, Takács N, Fernández de Mera IG, de la Fuente J, Farkas R (2014). Re-emergence of bovine piroplasmosis in Hungary: has the etiological role of *Babesia divergens* been taken over by *B. major* and *Theileria buffeli*?. Parasit Vectors.

[21] Hornok S, Estók P, Kováts D, Flaisz B, Takács N, Szőke K (2015). Screening of bat faeces for arthropod-borne apicomplexan protozoa: *Babesia canis* and *Besnoitia besnoiti*-like sequences from Chiroptera. Parasit Vectors.

[22] Hornok S, Kartali K, Takács N, Hofmann-Lehmann R (2016). Uneven seasonal distribution of *Babesia canis* and its two *18S* rDNA genotypes in questing *Dermacentor reticulatus* ticks in urban habitats. Ticks Tick Borne Dis.

[23] Márton M, Markolt F, Szabó L, Kozák L, Lanszki J, Patkó L (2016). Den site selection of the European badger, *Meles meles* and the red fox, *Vulpes vulpes* in Hungary. Fol Zool.

[24] Uspensky I (2002). Preliminary observations on specific adaptations of exophilic ixodid ticks to forests or open country habitats. Exp Appl Acarol.

[25] Hornok S, Farkas R (2009). Influence of biotope on the distribution and peak activity of questing ixodid ticks in Hungary. Med Vet Entomol.

[26] Földvári G, Farkas R (2005). Ixodid tick species attaching to dogs in Hungary. Vet Parasitol.

[27] Jefferies R, Ryan UM, Jardine J, Broughton DK, Robertson ID, Irwin PJ (2007). Blood, bull terriers and babesiosis: further evidence for direct transmission of *Babesia gibsoni* in dogs. Aust Vet J.

[28] Malagon F, Tapia JL (1994). Experimental transmission of *Babesia microti* infection by the oral route. Parasitol Res.

